# An Efficient Time-Varying Filter for Detrending and Bandwidth Limiting the Heart Rate Variability Tachogram without Resampling: MATLAB Open-Source Code and Internet Web-Based Implementation

**DOI:** 10.1155/2012/578785

**Published:** 2012-03-06

**Authors:** A. Eleuteri, A. C. Fisher, D. Groves, C. J. Dewhurst

**Affiliations:** ^1^Department of Medical Physics & Clinical Engineering, Royal Liverpool & Broadgreen University Hospital, Liverpool L7 8XP, UK; ^2^National Refractory Angina Centre, Royal Liverpool & Broadgreen University Hospital, Liverpool L7 8XP, UK; ^3^Department of Neonatal Medicine, Liverpool Women's Hospital, Liverpool L8 7SS, UK

## Abstract

The heart rate variability (HRV) signal derived from the ECG is a beat-to-beat record of RR intervals and is, as a time series, irregularly sampled. It is common engineering practice to resample this record, typically at 4 Hz, onto a regular time axis for analysis in advance of time domain filtering and spectral analysis based on the DFT. However, it is recognised that resampling introduces noise and frequency bias. The present work describes the implementation of a time-varying filter using a smoothing priors approach based on a Gaussian process model, which does not require data to be regular in time. Its output is directly compatible with the Lomb-Scargle algorithm for power density estimation. A web-based demonstration is available over the Internet for exemplar data. The MATLAB (MathWorks Inc.) code can be downloaded as open source.

## 1. Introduction

A time record consisting of beat-to-beat RR intervals is referred to as the heart rate tachogram. This forms the basis for a number of metrics of heart rate variability (HRV). The simplest measures of HRV are based on variance determined over a range of time periods. More complex measures can be derived from power spectrum density (PSD) estimations. The two most commonly used PSDs are the Welch Periodogram, based on the DFT, and the AR Spectrum, based on an autoregressive process model [1]. Both approaches require the data to be sampled regularly. Resampling the raw HRV data onto a regular time axis introduces noise into the signal and the information quality is compromised [1]. Conventionally, the HRV power is reported over 3 bandwidths: [0.01 ⋯ 0.04] Hz (Very Low Frequency, VLF) [0.04 ⋯ 0.15] Hz (Low Frequency, LF), and [0.15 ⋯ 0.4] Hz (High Frequency, HF) [1, 2].

Prior to transformation into the frequency domain, normal practice requires that the time series data are “detrended” or “high-pass filtered” at a very low frequency, say ~0.005 Hz. There is no universally formal justification for such detrending other than it minimises the effects of medium-term nonstationarity within the immediate time epoch (window) of interest [2]. Stationarity is an axiomatic assumption in conventional time-to-frequency transformation of the PSD (see Appendix B). 

A number of methods have been described to identify the trend component in the tachogram such that it can be simply removed by subtraction. These methods include fixed low-order polynomials [3, 4], adaptive higher-order polynomials [5, 6], and, more recently, the smoothing by priors approach (SPA) proposed by [7] which they describe as a time-varying finite impulse high-pass filter. The SPA uses a technique well-established in modern time series analysis and it addresses directly the phenomenon of nonstationarity.

However, the Tarvainen approach suffers two limitations. The first is conceptual: the algorithm requires resampling by interpolation onto a regular time axis. The second is practical: the MATLAB implementation is computationally inefficient and expensive and consequently very slow. In practice, its application is limited to relatively short tachograms [7]. 

In the present work, a novel algorithm is introduced which obviates these limitations by extending the SPA. The Smoothing by Gaussian process Priors (SGP) method described here explicitly does not require resampling and executes in MATLAB at least an order of magnitude faster than the SPA. By employing the SGP twice in sequence, the bandpass effect achieves detrending (high-pass) and low-pass filtering which is directly compatible with the Lomb Scargle Periodogram (LSP) [8]. 

## 2. The Smoothing Priors Approach

The SPA method considers the problem of modelling the trend component in a time series with a linear observation model:

(1) $$z_{\text{trend}}=Hy+v,$$

where **H** is the observation matrix, *v* is observation error, and *y* are parameters to be determined. The solution to estimating the trend is then expressed in terms of minimisation of a regularised least squares problem:

(2) $${\hat{y}}_{\sigma }=\underset{y}{\text{argmin}}{||Hy-z||}^{2}+\sigma^{2}{||D_{d}\left(Hy\right)||}^{2},$$

where *σ* is a regularisation parameter and **D**_*d*_ is the discrete approximation to the *d*th derivative operator.

By choosing **H** as the identity matrix, and *d* = 2, the solution can be written as

(3) $$\begin{array}{cc} {\hat{y}}_{\sigma } & ={\left(I+\sigma^{2}D_{2}^{\text{T}}D_{2}\right)}^{-1}z. \end{array}$$

Tarvainen et al. argue that selection of the observation matrix is done to simplify things, in the context of estimating parameters in a finite-dimensional space. A Bayesian interpretation of (2) is given, but always in the context of finite-dimensional parameter spaces. It is interesting and useful to give a different interpretation in the context of Gaussian Process (GP) priors, which implies a function-space view, rather than a parametric view, of the regression problem. In passing it is noted that the SPA, as published, is markedly inefficient and potentially unstable in using matrix inversion. A more efficient approach is presented as Appendix C. 

## 3. An Alternative Smoothing Prior Operator

Use of the **D**_2_ operator implies uniform sampling of the data and in the case of the HRV tachogram requires that the raw data be projected onto a regular time axis using some means of interpolation. Such a projection is frequently referred to as *resampling* which is undesirable in that it corrupts, preferentially, the higher frequency components [2]. In the present development, it is proposed that resampling can be avoided by using a different approximation for the second-order derivative operator. The usual approximation is based on a centred formula:

(4) $$f^{\prime \prime }\left(x_{i}\right)=\frac{f\left(x_{i+1}\right)-2f\left(x_{i}\right)+f\left(x_{i-1}\right)}{h^{2}}+O\left(h^{2}\right),$$

which implies that each row of the **D**_2_ matrix is the constant vector [1,−2,1]. 

A different approximation formula to the derivative, which does not imply uniform sampling, can also be obtained by Taylor expansion with nonuniform increments. After some algebra,

(5) $$\begin{array}{cc} f^{\prime \prime }\left(x_{i}\right) & =2\frac{f\left(x_{i+1}\right)\left(x_{i-1}-x_{i}\right)-f\left(x_{i}\right)\left(x_{i-1}-x_{i+1}\right)+f\left(x_{i-1}\right)\left(x_{i}-x_{i+1}\right)}{\left(x_{i-1}-x_{i}\right)\left(x_{i-1}-x_{i+1}\right)\left(x_{i}-x_{i+1}\right)}+O\left(h\right), \end{array}$$

where *h* is now the maximum local grid spacing.

The rows of the operator now explicitly depend on the *x* values as desired:

(6) $$\;\begin{array}{cc} & [\frac{2}{\left(x_{i+1}-x_{i-1}\right)\left(x_{i+1}-x_{i}\right)},\;\\ & \;-\frac{2}{\left(x_{i+1}-x_{i}\right)\left(x_{i}-x_{i-1}\right)},\frac{2}{\left(x_{i+1}-x_{i-1}\right)\left(x_{i}-x_{i-1}\right)}]. \end{array}$$

The operator is denoted by the symbol ${\hat{D}}_{2}$.

An efficient implementation of the above algorithm (MATLAB) is the following:

T = length(x);
id = 2 : (T − 1);
idp1 = id + 1;
idm1 = id − 1;
V1 = 2./((x(idm1) − x(idp1)). *(x(id) − x(idp1)));
V2 = −2./((x(idm1) − x(id)). *(x(id) − x(idp1)));
V3 = 2./((x(idm1) − x(id)). *(x(idm1) − x(idp1)));
D2hat = spdiags  ([V1, V2, V3]∖V1(1), [0 : 2], T − 2, T);
L = chol(speye(T) + sigma ^∧^2 *D2hat*'* *D2hat, ‘lower');
*z*_stat = *z* − L*'*∖(L∖*z*);

Note that to reduce the possibility of numerical instabilities in the solution of the linear systems, the D2hat matrix is normalised by the first element of vector V1. 

## 4. Equivalent Kernel and Smoothing

The operation of the smoothing priors can be understood by looking at the following simplified form:

(7) y=Hz,

where *z* is the vector of data and **H** is the matrix coefficient of (3). The smoother acts as a linear filter.

Since each element of *z* and *y* can be thought of as placed at a distinct time point, it is seen that each row of the **H** matrix acts over all the elements of *z* to produce a single element of *y*. Consequently, the filter is noncausal. In fact, each row of **H** defines a *weighting function*. Each weighting function is localised around a specific time, and its bandwidth determines how many samples from the past and from the future contribute to the estimate. The wider the weighting function, the smoother the resulting estimates.

**Figure 1:**
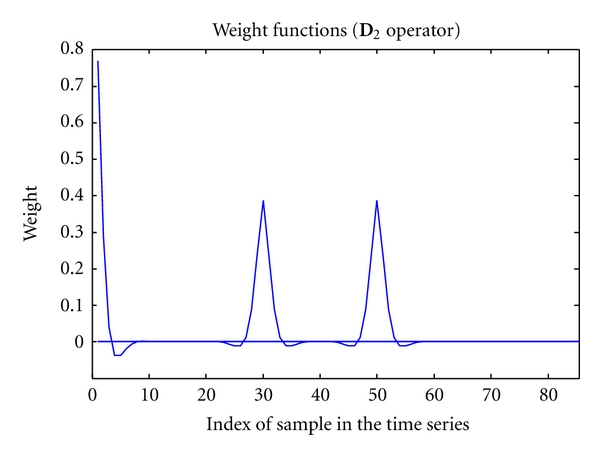
Weight functions (*viz.* **D**_2_ operator).

In the case of uniformly sampled data, the weighting functions have the same shape (except at the boundaries), which can be imagined as a sliding window translating in time: this is a consequence of the definition of the **D**_2_ operator, which is time independent.  Figure 1 shows some weight functions implied by the **D**_2_ operator.

However, for the case of arbitrarily (irregularly) sampled data of the HRV tachogram, the ${\hat{D}}_{2}$ operator actually depends on time; therefore the weighting functions will take on a different shape. This makes the resulting filter effectively a time-variant filter. It is possible to calculate the transfer function of the filter **H** in the limit as the number of data points tends to infinity. It can be shown [2] that the (non-stationary) spectral density of the Gaussian process prior is

(8) $$S\left(f\right)\propto \frac{1}{4\pi^{2}f^{4}}.$$

From the above, the power spectral density of the equivalent kernel filter is derived as

(9) $$h\left(f\right)=\frac{1}{\sigma^{2}4\pi^{2}f^{4}+1}.$$

**Figure 2:**
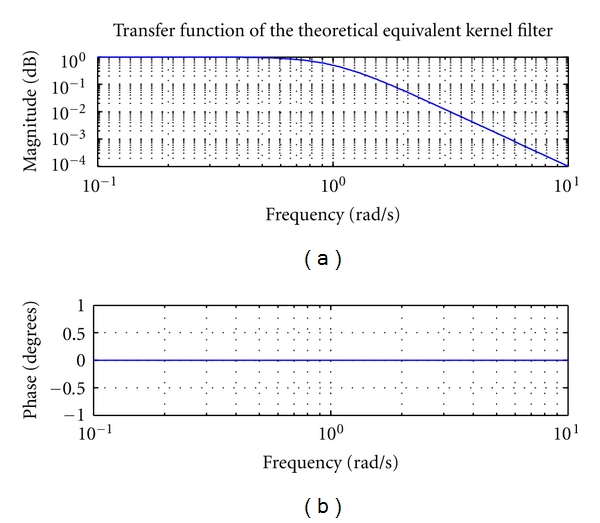
Bode plot of theoretical transfer function of equivalent kernel filter.

In Figure 2 it is shown an example of the transfer function of the equivalent kernel filter (with *σ* ^2^ = 1): the phase is constant zero.

## 5. Estimation of the Filter Bandwidth

Although the approximation in (9) is only valid in the limit as the number of data points goes to infinity, it is still useful for calculating the approximate −3 dB bandwidth of the finite-sample approximation of the filter in terms of the smoothing parameter *σ* ^2^. Whereas the SPA as presented [7] does not provide an effective bandwidth estimate but only the qualitative behaviour of the filter, the following approximation provides a quantitative tool.

Inverting (9) and applying the bilinear transformation of the continuous frequencies, we get

(10) $$\sigma^{2}=\left(\sqrt{2}-1\right){\left(2\tan\left(\frac{\omega_{c}\pi }{2}\right)\right)}^{-4},$$

where *ω*_*c*_ is the normalised cut-off frequency (namely, the Nyquist frequency = 1).

Since the number of data points mostly impacts the estimation of low frequencies, the expectation is that the approximation is good in the low-frequency range.

In a Monte Carlo simulation, 1000 replications of the Welch periodogram estimates were made of white Gaussian noise coloured through the equivalent filter **H**. Each noise sequence was composed of 5000 regularly spaced samples. In Table 1, it is seen that this approximation is good and, predictably, deteriorates as the cut-off frequency increases.

**Figure 3:**
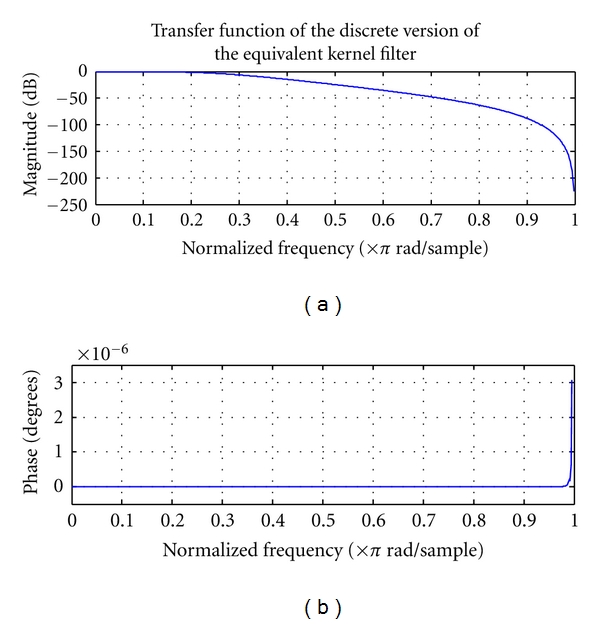
Bode plot of discrete transfer function of equivalent kernel filter.

Figure 3 shows the transfer function of the digital equivalent kernel filter.

There is very little phase distortion, except at very high frequencies close to the Nyquist frequency.

## 6. Illustrative Performance with Synthetic and Real Data Sets

A synthetic data set of was generated (MATLAB) as series of normallydistributed random numbers of mean 0.85(1) s (equivalent to a heart rate of ~75 bpm) and std 0.025 s: this was low-pass filtered at 1 Hz (3rd-order phase-less IIR). These data were projected by interpolation, onto an irregular time axis of mean interval 0.86(1) s and variance 0.01s^2^. The resulting synthetic HRV record, as a time record of band-limited Gaussian noise, was of 30 s duration, average sampling frequency of 1.15(6) Hz and had no significant power above 1 Hz.

Clinical ECG data from a Lead II configuration were recorded from a healthy adult seated for a period of 60 minutes using a Spacelabs Medical Pathfinder Holter system. RR intervals were available with 1 ms resolution.

**Figure 4:**
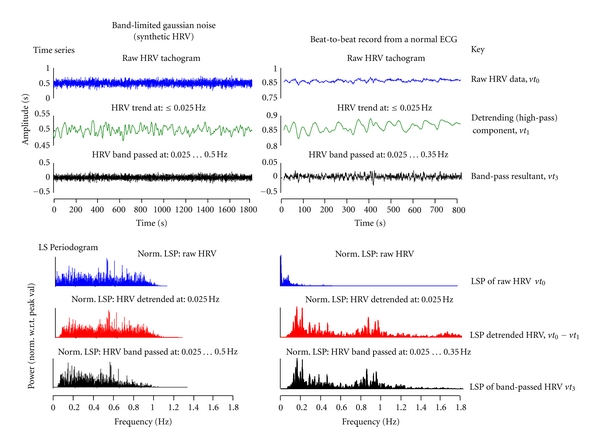
Synthetic and clinical HRV records band-pass filtered by sequential application of SGP: raw data *vt*_0_ “smoothed” to give *vt*_1_; *vt*_2_ = *vt*_0_ − *vt*_1_ (not shown); *vt*_2_ “smoothed” to give *vt*_3_. Lomb Scargle Periodograms (LSPs) are for *vt*_0_, *vt*_2_, and *vt*_3_.

The time domain and frequency domain (as the Lomb Scargle periodogram) representations of the synthetic data set and the clinical data set are shown in Figure 4 to illustrate the band-pass filtering effect achieved using sequential SGP. The synthetic HRV data and the clinical HRV data are filtered in the band-pass [0.025 *⋯* 0.5] Hz and [0.025 *⋯* 0.35] Hz, respectively.

## 7. Internet Resources and Open-Source Code

Resources relevant to this work are located at http://clinengnhs.liv.ac.uk/links.htm and include the following.

1. A website demonstration of SGP running on an automation instance of MATLAB 2008a. Developed for JavaScript-enabled MS IE6+ and FireFox browsers.
2. MATLAB open-source code:
  - Smoothing by Gaussian process Priors (SGP): gpsmooth_3.m,
  - Optimized Lomb Scargle Periodogram (fLSPw:* fastest Lomb Scargle Periodogram in the West*): fLSPw.m.

## 8. Conclusion

The SGP (Smoothing by Gaussian process Priors) algorithm is a second-order response time-varying filter which operates on irregularly sampled data without compromising low-frequency fidelity. In the context of Heart Rate Variability analysis, it provides detrending (high-pass) and low-pass filtering with explicitly specified −3 dB cut-off points. A small limitation is the implicit requirement to assume a *representative* sampling frequency to establish the frequency interval: here this is taken as the reciprocal of the median sampling interval. The SGP MATLAB code is available as open source via a comprehensive website and is directly compatible with an optimised implementation of the Lomb Scargle Periodogram (fLSPw) estimator.

## Figures and Tables

**Figure 5:**
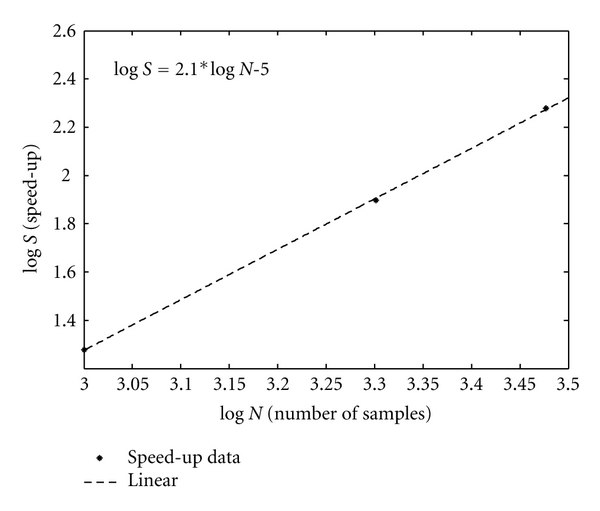
Speed-up of SGP over SPA with increasing data set size.

**Table 1:** approximation of −3 dB point [Hz].

True –3 dB cut-off frequency	Approximate frequency
0.05	0.049
0.1	0.102
0.2	0.208
0.3	0.34

## Appendices

### A. Gaussian Process Interpretation of Smoothing Priors

Consider the posterior expectation of a GP regressor (2) at a set of training data points *z*:

(A.1) $${\hat{y}}_{\sigma }=K{\left(K+\sigma^{2}I\right)}^{-1}z,$$

where **K** is the covariance matrix of the GP *y* and *σ* is the standard deviation of the white (Gaussian) noise corrupting the data *z*. By algebraic manipulation of (A.1), it follows:

(A.2) $$\begin{array}{cc} {\hat{y}}_{\sigma } & ={\left[\left(K+\sigma^{2}I\right)K^{-1}\right]}^{-1}z\equiv {\left(KK^{-1}+\sigma^{2}K^{-1}\right)}^{-1}z\\ & \equiv {\left(I+\sigma^{2}K^{-1}\right)}^{-1}z. \end{array}$$

Comparing the above with (3),

(A.3) $$D_{d}^{\text{T}}D_{d}=K^{-1}.$$

The above derivations show some important facts about the solution of the problem.

1. The parameter *σ* describes the amount of (Gaussian) white noise, which affects the data. As *σ* gets smaller, the filtering process gets smoother.
2. The smoothness properties of the resulting estimator depend not only on *σ*, but also on the choice of the covariance matrix **K**. Note that polynomials *up to* (and including) 1st degree are in the null space of the regularization operator (i.e., they are both mapped to constants), which means that they are not penalized at all. This implies that the Gaussian Process prior is not stationary (see Appendix B for a definition).

### B. Stationarity

A Gaussian process is completely described by its mean function and covariance function. Given a real process *f*(*t*), these functions are specified as the following expectations:

(B.1) $$\begin{array}{c} m\left(t\right)=\text{E}\left[f\left(t\right)\right],\\ k\left(t,t\prime \right)=\text{E}\left[\left(f\left(t\right)-m\left(t\right)\right)\left(f\left(t\prime \right)-m\left(t^{\prime }\right)\right)\right]. \end{array}$$

For a fixed *t*, *f*(*t*) is a Gaussian random variable with mean *m*(*t*) and variance *k*(*t*, *t*), so that a Gaussian process can be defined as a collection of random variables, any finite number of which have a joint Gaussian distribution.

A stationary covariance function is a function of *t* − *t'*, that is, it is invariant to translations. The above definitions can be used to define stationarity for Gaussian processes. A process which has constant mean and whose covariance function is stationary is called *weakly stationary *(or* wide-sense stationary, WSS*). A process whose joint distributions are invariant to translations, that is, the statistics of *f*(*t*) and *f*(*t* + *c*) are the same for any *c*, is called *strictly stationary *(or* strict-sense stationary, SSS*). It can be shown that as SSS process is also WSS, and if the process is Gaussian, then the converse is also true.

If any of the above conditions are violated, then the process is non-stationary; an example is the Gaussian process whose inverse covariance matrix is given by (4) and (5).

### C. Improving the Speed and Stability of the SPA Smoothing Process

In general, matrix inversion is very computationally expensive and should be avoided whenever possible A more efficient solution uses the *backslash* operator ∖, which in MATLAB implements the solution of a linear system by Gaussian elimination. However, the matrix (**I** + *σ* ^2^ **D**_2_^T^ **D**_2_) can be nearly singular and ill conditioned, depending on values of the parameter *σ* ^2^. To circumvent this risk, the lower Cholesky factor **L** (the *square root*) of this matrix is derived, so that

(C.1) $$LL^{\text{T}}=\left(I+\sigma^{2}D_{2}^{\text{T}}D_{2}\right).$$

With this decomposition, matrix inversion can then simply be written as the solution, in sequence, of two triangular systems of linear equations, which is a very fast and numerically stable operation:

(C.2) $${\hat{y}}_{\sigma }=L^{\text{T}}\setminus \left(L\setminus z\right).$$

Although the theoretical computational complexity of straight matrix inversion and the above (seemingly more complex) steps is the same, the *hidden factors* of the actual numerical computations make a very significant difference [9]. The speed-up is illustrated by performing the above computations on a sequence of varying length (from 1000 to 3000 samples), repeating the execution of both algorithms 100 times.  Figure 5 shows the speed-up as a function of the data set size.

It is clear that, as the dimension of the data set increases, the speed-up increases quadratically, showing the inefficiency of the matrix inversion-based smoothing. The following code (MATLAB R006b) was used:

T = length(*z*);
D2 = spdiags(ones(T − 2,1) *[1 − 2  1, 0 : 2], T − 2, T);
L = chol(speye(T) + sigma ^∧^2 *D2*'* *D2, ‘lower'); %  warning:  potential  bottleneck!
*z*_stat = *z* − L*'*∖(L∖*z*);

It should be noted that in MATLAB R2006a, and possibly previous versions, multiplication of the *σ* ^2^ coefficient by the sparse matrix is anomalously a very slow operation.
